# Systematic Review of Functional Mapping and Cortical Reorganization in the Setting of Arteriovenous Malformations, Redefining Anatomical Eloquence

**DOI:** 10.3389/fsurg.2020.514247

**Published:** 2020-09-30

**Authors:** Sauson Soldozy, Daniel K. Akyeampong, David L. Barquin, Pedro Norat, Kaan Yağmurlu, Jennifer D. Sokolowski, Khadijeh A. Sharifi, Petr Tvrdik, Min S. Park, M. Yashar S. Kalani

**Affiliations:** Department of Neurological Surgery, University of Virginia Health System, Charlottesville, VA, United States

**Keywords:** arteriovenous malformation (AVM), functional reorganization, brain mapping, neurosurgical outcomes, fMRI, TMS (transcranial magnetic stimulation), MEG (magnetoencephalography), MSI (magnetic source imaging)

## Abstract

**Objective:** The goal of this study was to systematically review functional mapping and reorganization that takes place in the setting of arteriovenous malformations (AVMs) and its potential impact on grading and surgical decision making.

**Methods:** A systematic literature review was performed using the PubMed database for studies published between 1986 and 2019. Studies assessing brain mapping and functional reorganization in AVMs were included.

**Results:** Of the total 84 articles identified in the original literature search, 12 studies were ultimately selected. This includes studies evaluating the impact of cortical reorganization on patient outcomes and factors impacting and triggering cortical reorganization in AVM.

**Conclusion:** These studies demonstrate the utility of preoperative brain mapping and acknowledgment of functional reorganization in the setting of AVMs. While these findings led to alterations in Spetzler–Martin grading and subsequent surgical decision making, it remains unclear the clinical utility of this information when assessing patient outcomes. While promising, more research is required before recommendations can be made regarding functional brain mapping and cortical reorganization with respect to AVM surgery involving eloquent brain tissue.

## Introduction

Functional reorganization refers to a change in the size or location of task-specific neural processing centers. Identifying cortical reorganization has been made possible by the advent of brain mapping techniques, with evidence of reorganization taking place in neurodegenerative disease, stroke, and brain tumors (1). This includes functional magnetic resonance imaging (fMRI), positron emission tomography (PET), magnetoencephalography (MEG)/magnetic source imaging (MSI), and transcranial magnetic stimulation (TMS), which have enabled mapping of cortical architecture in real time. These modalities have also proved useful in surgical planning and resection of lesions surrounding eloquent anatomy. Limiting postoperative deficit and mortality, cortical mapping has been shown to improve outcomes in the treatment of gliomas and other brain tumors (1, 2).

With respect to arteriovenous malformations (AVMs), however, there is limited data with respect to the implementation of brain mapping to assess for functional reorganization and its impact on patient outcomes. AVMs are considered congenital lesions that are likely present prior to the maturation of eloquent areas, suggesting that patients have an increased susceptibility to functional displacement given the inherent plasticity of the cortex during growth and development (3, 4). Cortical plasticity is thought to be further augmented in AVMs by chronic hypoperfusion due to vascular steal, resulting in translocation of neurological functions to other brain regions leaving nonfunctional tissue remaining within the AVM site (4, 5). The first mention of functional cerebral imaging being used in the setting of AVMs was more than two decades ago, where it was determined that PET imaging was a reliable method in evaluating the relationship of motor, language, and visual cortex centers with AVMs (6); additionally, given the lack of mass effect and cerebral swelling, AVMs were determined to be good candidates for functional imaging, as there is minimal brain distortion confounding imaging interpretation when compared with other lesion types (6–8), although the effects of cerebrovascular reactivity and presence of hemosiderin inherent to AVMs make non-blood flow-based techniques particularly important (9).

With advancements in modern neuroimaging permitting mapping of functional connectivity, eloquent brain anatomy may be better delineated in the preoperative planning stages of AVM surgery. In this systematic review, the authors identify and review studies presenting the use of brain mapping in the setting of AVMs. The implications of preoperative mapping and presence of functional reorganization are discussed in the context of grading and surgical decision making.

## Methods

A literature search using PubMed and MEDLINE databases was performed following PRISMA guidelines (10). Using common evidence medicine framework PICO (Patient Population, Intervention, Control, Outcome), we defined our research question: What is the impact of functional mapping and cortical reorganization on surgical decision making in patients undergoing treatment for AVM? No current review protocol exists. The following three search queries were performed without language restriction to identify all relevant titles and abstracts in the NCBI/NLM PubMed and Medline databases: (arteriovenous malformation OR AVM) AND (functional OR cortical reorganization), (arteriovenous malformation OR AVM) AND (transcranial magnetic stimulation OR TMS), (arteriovenous malformation OR AVM) AND (magnetoencephalography OR MEG).

### Inclusion Criteria

In order to meet inclusion criteria, studies must utilize fMRI, PET TMS, or MEG/MSI techniques in the setting of AVM resection or radiosurgery. Articles evaluating proximity to eloquent anatomy, cortical reorganization, brain mapping, surgical planning, and outcomes in the setting of AVM resection, embolization, or radiosurgery were included.

### Exclusion Criteria

The exclusion criteria were editorials, letters, reviews without original data, case reports with less than five patients, and studies evaluating other brain lesions including but not limited to cavernous malformation, dural arteriovenous fistula, glioma, and meningioma. The reference lists of each included article were surveyed based on these inclusion/exclusion criteria.

## Results

**Figure 1:**
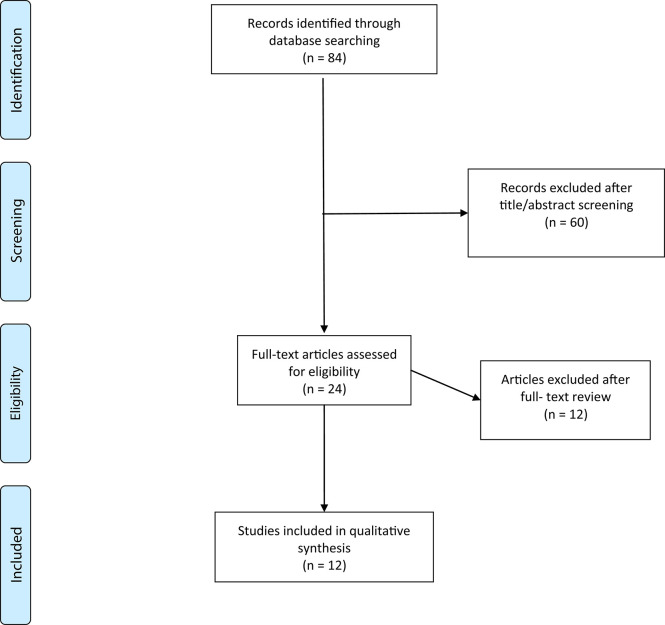
Flow diagram showing process of study selection.

The Medline search returned 30 articles with the term (arteriovenous malformation OR AVM) AND (functional OR cortical) organization, 18 articles with the term (arteriovenous malformation OR AVM) AND (transcranial magnetic stimulation OR TMS), and 36 articles with the term (arteriovenous malformation OR AVM) AND (magnetoencephalography OR MEG), yielding a total of 84 articles. After the titles and abstracts were reviewed for relevance, a total of 24 articles were selected. After a full-text review based on exclusion criteria, 12 articles remained and were included for qualitative analysis. A PRISMA flow diagram detailing methods for selection of studies is presented in Figure 1. Data from the included studies were independently reviewed and extracted by the first, second, and third authors. The studies are organized based on the modality of brain mapping used (Tables 1, 2).

**Table 1:** Summary of studies and findings utilizing blood-based mapping.

**Study**	**AVM Patients (n)**	**Study type**	**Follow-up**	**Mean SM Grading (*n*)**	**Cortical reorganization occurrence (*n*, %)**	**Study Conclusions**
(11)	43	Retrospective	1–2 weeks 6–30 months	2.98	18, 41.9%	Although right-sided lateralization was identified preoperatively in some patients, follow-up language evaluation did not identify a decreased incidence of language dysfunction in this cohort after surgery
(12)	48	Retrospective	1 week 6 months	3.04	11, 22.9%	Cortical reorganization was not statistically different among patients with and without motor strength worsening in both follow-up periods
(13)	15	Retrospective	1 week 6 months	3.2	6, 40%	There were no statistically significant differences in cortical reorganization between good and poor outcome groups
(3)	22	Retrospective	44 months		6, 27.3%	Cortical reorganization takes place when an AVM is directly overlying the motor cortex. This suggests potentially safe resection of AVM from eloquent brain regions.
(14)	11	Retrospective	4–24 months		5, 45.5%	The presence and degree of cortical reorganization appears to be task and lesion location dependent, and that based on postoperative follow-up fMRI, LI change in lesions were modifiable by severe flow changes making lateralization interpretation difficult in those lesions with flow abnormalities.
(15)	9	Prospective			4, 44.4%	Functional reorganization was shown to occur in the setting of AVM but was not based on distortion of anatomy. Three distinct patterns were observed but these were not predictable. AVM hemodynamics may confound fMRI interpretation, although this remains unclear.

*n, sample size patients with arteriovenous malformation; AVM, arteriovenous malformation; GTR, gross-total resection; fMRI, functional magnetic resonance imaging; LI, lateralization index*.

**Table 2:** Summary of studies and findings utilizing nonblood-based mapping.

**Study**	**AVM Patients (n)**	**Study type**	**Follow-up**	**Mean SM Grading (n)**	**Cortical reorganization occurrence (%)**	**Study Conclusions**
(16)	10	Retrospective	3 months	2.5	6, 60%^*^	Brain organization is a dynamic process based on continuously varying cortico-subcortical networks, and nTMS permits greater accuracy in defining eloquence that may impact SM grading and patient outcomes.
				2.2 (nTMS)		
(17)	34	Prospective	5 days 3 months	2.38 (nTMS)	9, 26.5%^*^	nTMS mapping is a valid surgical planning tool that impacts SM grading and therefore treatment recommendations and outcomes.
(18)	10	Prospective		2.6	1, 10%	Cortical mapping with nTMS is especially useful in the event anatomical eloquence cannot be identified due to mass effect distortion of hypervascularization, providing information useful for AVM treatment
(19)	5	Retrospective	6, 12, 24, and 36 months	3.2	1, 20%	MEG mapping is a useful tool in Gamma Knife dose selection and isocenter placement planning for AVMs located in eloquent brain cortex.
(20)	30	Retrospective	9–66 months	3.13	10, 33.3%	Preoperative MSI may assist in improving AVM treatment strategies by helping to visualize the relationship of the sensorimotor cortex with respect to nearby AVM, although presence of hemispheric shift did not correlate with outcome.
(21)	8	Prospective	12 months			Magnetoencephalographic angiography is a promising technique that aids in risk stratification and selective vessel embolization

* *Determined based on number patients with modified SM grading due to altered eloquence scoring after nTMS mapping*.

### Functional MRI

Whereas traditional MRI is often utilized to understand anatomical relationships between brain structures, functional MRI is able to provide information regarding brain activity. This is useful to map out the spatiotemporal distribution of neuronal activation either in resting state or in response to stimuli.

In a study of 43 right-handed patients, Liu et al. (11) retrospectively analyzed postoperative language function in patients with AVMs located in the left hemisphere involving the language cortex. AVM involvement of Broca's area (inferior frontal and middle frontal gyri, including Brodmann areas 44, 45, 9, and 46) was present in 13 patients, with 30 patients having involvement of Wernicke's area (supramarginal, angular, and superior temporal gyri, including Brodmann areas 22, 21, 39, and 40). Hemorrhage occurred in 17 (39.5%) patients, seizures in 15 (34.9%), and headache in 6 (20.0%) patients. Based on Spetzler–Martin (SM) grading, 7 patients demonstrated grade II AVMs, 30 patients grade III AVMs, and 6 patients grade IV. fMRI was performed 1–2 weeks prior to surgery, while patients were asked to perform language-based tasks during image acquisition. Postoperative fMRI was performed 1–2 weeks (short-term) after surgery as well as 6–30 months (long term) after surgery. In addition, Western Aphasia Battery assessment was performed both preoperatively and postoperatively. Language lateralization was quantified by the lateralization index (LI), where LI = (VL – VR)/(VL + VR), with VL and VR denoting left and right hemispheric voxel activation, respectively. A total of 18 (41.9%) patients were identified to have right-sided language lateralization (R group), with 25 (58.1%) patients being classified as have no right-sided lateralization (NR group), 22 of whom exhibited left-sided lateralization and 3 with no discernable hemispheric lateralization. No statistical differences were identified between these two groups with respect to gender (*p* = 0.501), age (*p* = 0.926), lesion location (*p* = 0.332), lesion size (*p* = 0.062), or SM grading (*p* = 0.386). Gross total resection was achievable for all lesions utilizing intraoperative neuronavigation. On short-term follow-up, language deterioration occurred in 7 (38.9%) patients in the R group, and in 11 (44.0%) patients the NR group, although this difference was not statistically significant (*p* = 0.738). On long-term follow-up, three (16.7%) patients and four (16%) patients continued to have persistent aphasia in the R and NR group, respectively (*p* = 1.000).

Lin et al. (12) retrospectively analyzed 48 patients with AVMs involving the primary and secondary motor cortex, corona radiata, and posterior limb of the internal capsule in an attempt to assess the efficacy of fMRI navigation in brain AVM surgery. The mean SM grade was 3.04, with a mean nidus size of 4.2 cm. Deep draining vein was present in four (8.3%) patients. Functional imaging was performed 1 week prior to surgery, with repetitive finger-to-thumb opposition movements or flexion–extension movements of the foot being used as motor stimulation paradigms. By measuring the number of activated voxels, cortical motor reorganization was determined when the extent of voxel activation in other brain regions was equal to or greater than that occurring in the functional location. Based on these criteria, 11 (22.9%) patients were determined to have undergone cortical reorganization, leaving 37 (77.1%) patients having no radiological evidence of reorganization. Postoperative muscle strength assessment was performed at 1 week and 6 months after surgery. Worsened muscle strength 1 week after surgery was observed in 21 (43.8%) patients, with 10 (20.8%) patients continuing to experience decreased muscle strength at 6 months' follow-up. On short-term and long-term follow-ups, cortical reorganization only trended toward significance between these two groups (*p* = 0.063 and 0.063, respectively).

In another study by Lin et al. (13), 15 patients with AVMs involving the hand motor cortex (precentral knob) and/or foot motor cortex (paracentral lobule) were retrospectively analyzed. Repetitive finger-to-thumb opposition movements or flexion–extension movements of the foot were used as motor paradigms, and cortical reorganization was based on motor activation in other areas as in the previous study (12). Each motor function was analyzed as an independent object, with a total of 18 objects being created and analyzed. For instance, concurrent involvement of hand and foot motor cortices in a single patient constituted two objects. The average SM grading was 3.17 ± 0.5, with preoperative muscle strength being intact in 17 (94.4%) objects. Thirteen (72.2%) objects exhibited hand motor cortex involvement, while five (27.8%) objects represented foot motor cortex involvement. Among these objects, eight (44.4%) demonstrated cortical reorganization on preoperative fMRI. Muscle strength was intact in 6 (33.3%) objects at 6 months, with 12 (66.7%) objects having a deficit at this follow-up time. Cortical reorganization was present in three (37.5%) of the intact objects at 6 months, and it was present in five (62.5%) objects who presented with a deficit at 6 months (*p* = 1.000).

Lee et al. (3) conducted a retrospective analysis of 22 patients with AVMs. Patients were divided into three groups according to lesion location. Group 1, the control group, included 13 (59.1%) patients whose AVM was greater than two gyri or sulci away from motor cortices. Group 2 comprised three (13.6%) patients with an AVM adjacent to motor areas, defined as being between 1 and 2 sulci or gyri away. Six (27.3%) patients were included in group 3, which constitutes an AVM directly overlying the motor areas. A motor finger-tapping paradigm for the upper limb and/or knee-flexion paradigm for the lower limb was implemented during fMRI sequence acquisition. Cortical reorganization was only observed in group 3 patients, and it was statistically significant when compared with group 1 patients (*p* = 0.00037). In group 3 patients, excision took place in three (50%) patients. Mean follow-up for those patients having undergone excision was 44 months, with no new or worsening of previous neurological deficits being noted after intervention.

Lehéricy et al. (14) published a study in which 11 patients with left-hemisphere brain AVMs and 10 control subjects were examined with fMRI while performing three different tasks: semantic fluency, covert sentence repetition, and story listening. Patients and control subjects were all right-handed. The prefrontal, anterior temporal, precentral, insula, inferior temporal, temporoparietal, and central cortices were among the lesion locations. LI, as described by Liu et al. (11) was utilized to determine hemispheric lateralization. Patients were divided into two groups. Group 1 comprised six (54.5%) patients who had LIs similar to those of the control subjects, whereas group 2 had five (45.5%) patients with LIs outside the range of the control subjects (mean ± 2 SDs). Cortical reorganization differed significantly between groups 1 and 2 based on the functional task at hand and cortex location. Temporal LIs in group 1 were significantly greater than those in group 2 (*p* < 0.05, Kruskal–Wallis test) during the story listening task. All other LIs were higher in group 1 than in group 2, but these differences did not reach statistical significance. In this study, embolization was the preferred treatment option, with four patients undergoing only embolization, four patients undergoing both embolization and surgery, two patients undergoing embolization and radiosurgery, and one patient having only surgery performed. Postoperative language function worsening occurred in three patients, with one patient being in group 1 and the other two patients being in group 2. Follow-up imaging was performed in group 2 patients 4–24 months after endovascular treatment, demonstrating postoperative hemispheric dominance to remain unchanged in one patient, changing from strongly right-sided to symmetric and weakly right-sided in one patient, and from strongly right-sided in both frontal and temporal lobes to symmetric in the temporal lobes and slightly left sided in the frontal lobes after treatment.

In a prospective study by Alkadhi et al. (15), fMRI was used to map the cortical organization in nine right-handed patients with brain AVMs directly involving the hand or foot region of the primary motor cortex (M1). These subjects were referred for endovascular treatment (*n* = 5) or for follow-up after partial embolization (*n* = 4). Two patients experienced transient mild hemiparesis postoperatively that had resolved completely. Of the nine patients, there were six (66.7%) with AVMs involving the M1 hand area and three (33.3%) with AVMs involving the M1 foot area. Cortical hand and foot mapping were done by asking patients to perform simple, self-paced movements at a constant rhythm of ~1 cycle per second, including opening and closing the hand or making a flexion–extension movement of the foot. Hemodynamic differences between the AVM and adjacent healthy tissue were accounted for by reprocessing the data with different signal models. The study determined that it is possible for AVM hemodynamics to impede blood oxygen level-dependent (BOLD) signaling, potentially leading to inaccurate functional mapping. Activation within the affected hemisphere was compared with the unaffected hemisphere with somatotopic maps obtained from fMRI. In the six patients with AVMs involving the M1 hand area, four (66.7%) subjects showed functional displacement within the affected M1 contralateral to the moving hand with two of these patients showing additional activation in the ipsilateral M1. Overall, three organization patterns were observed: (1) functional displacement within the M1 cortex, (2) hemispheric shift, and (3) function taken over from nonprimary motor areas. Distortion of the anatomy caused by the AVMs was not shown to influence the location of the reorganized cortex in these four subjects.

Based on the findings of these studies, fMRI is able to detect cortical reorganization in AVM patients, occurring in 20–40% of patients with eloquent brain AVM. However, the detection of cortical reorganization does not appear to confer statistically significant improvement in outcomes, with patients with or without reorganization experiencing similar deficit rates.

### Transcranial Magnetic Stimulation

Noninvasive brain stimulation is achievable with the use of TMS, which produces a high-intensity magnetic field to either excite or inhibit underlying brain tissue. This is useful for both generating evoked potentials and mapping sensory and motor cortex.

Germanò et al. (16) in a recent study, utilized navigated TMS (nTMS) to identify eloquent brain regions in relation to nearby AVMs in 10 patients. Of these 10 patients, 4 (40%) presented with ruptured AVMs and were symptomatic, although a single patient with an unruptured AVM experienced preoperative right motor weakness. By combining nTMS somatotopic mapping with diffusion tensor imaging–fiber tracking (DTI-FT), three-dimensional visualization of motor, speech, and visual pathways was performed to more accurately define and score eloquence when determining SM grading. A variation in SM grading was noted in six (60%) patients after eloquence was scored based on nTMS, with five patients showing a decrease in grading from grade II to I and one patient increasing from grade II to III when compared with eloquence scoring by MR scan. The difference in eloquence grading between nTMS mapping and brain MR approached statistical significance (*p* = 0.05). Post-treatment neurologic assessment was either unchanged (*n* = 7) or improved (*n* = 3) on 3-month follow up.

Ille et al. (17) published similar findings to Germanò et al. (16) showing that nTMS mapping resulted in modified SM grading of AVMs. By combining nTMS and nTMS-based DTI-FT, white matter tracts and motor and speech networks were visualized in a total of 34 patients. Preoperatively, four patients presented with neurologic deficit. nTMS mapping changed SM grading in nine (26.5%) cases. Of these cases, six (17.6%) were changed to a lower grade, whereas three (8.8%) changed to a higher grade. Decision making with respect to microsurgical resection was impacted by nTMS mapping in 21 (61.8%) cases. The decision to not undergo surgery was due to nTMS mapping in all cases. On follow-up, transient deficits were experienced by 6 (17.6%) patients, permanent deficits in 3 (8.8%) patients, and no change in the remaining 25 (73.5%) patients.

Kato et al. (18) performed preoperative functional mapping of both primary motor cortex and speech related areas in 10 patients with unruptured AVMs. Six (60%) patients underwent motor mapping with AVMs near the Rolandic region, with speech mapping being performed in the remaining four (40%) patients with left perisylvian AVMs. The primary cortex was easily identified with nTMS despite disruption of anatomical landmarks by surrounding AVM vasculature. Motor cortex reorganization was not observed, although a single case (10%) of right hemispheric language dominance was observed in a right-handed patient with a left frontal AVM, indicating a hemispheric shift.

The utilization for nTMS has the potential to impact patient care with respect to modifying SM grading based on redefined anatomical eloquence. In the aforementioned studies, SM grading was often downgraded, impacting the decision-making process with respect to pursuing surgery or not. Further validation in large prospective studies is necessary before nTMS can be recommend for routine application.

### Magnetoencephalography/Magnetic Source Imaging

Rather than using magnetic fields to stimulate brain tissue like in TMS, magnetoencephalography (MEG), or MSI, records magnetic fields produced by the electrical activity of neurons. Through magnetometers, MEG is another method by which physicians can map cortex prior to surgery.

Utilizing MEG, Bowden et al. (19) mapped the motor cortex of five patients with AVMs treated with stereotactic radiosurgery (SRS). MEG analyses were coregistered with high-resolution MR images to produce functional brain maps, which are then integrated with intraoperative stereotactic MRI used to create an SRS planning model. No patients had neurological deficit prior to SRS. In one (25%) patient, MEG analysis demonstrated right-hand motor function to be present bilaterally, indicating an AVM-induced plastic change. This led to a reduced margin dose and removal of an isocenter, and applying blocking such that dosing to the gyrus immediately anterior to the AVM was reduced. In the other patients, MEG also contributed to preoperative SRS planning to spare adjacent tissues. Two patients (40%) experienced reduced seizure frequency and severity, and one (25%) patient who was SM grade IV suffered from left hemiparesis due to hemorrhage 7 months postoperatively.

In a retrospective study utilizing MSI, Vates et al. (20) analyzed 30 patients with AVMs, 14 (47%) of whom had AVMs within the primary cortex, including six patients with transrolandic AVMs, five with parietal lobe AVMs, and three with frontal lobe AVMs. These mapping data correlated to higher SM grading performed prior to MSI. A total of 10 patients (33%) demonstrated cortical plasticity in the somatosensory homunculus. Among these, in three patients, a complete hemispheric shift was noted, with more subtle translocations to adjacent gyri being noted in the remaining seven patients. Patients with direct involvement of the primary cortex had worse outcomes than had those patients with AVMs sparing the primary cortex (Mann–Whitney *U*-test, *z* = −2.3, *p* = 0.02). In addition, patients experiencing cortical shift fared no better than those in which shift was absent, with no correlation between shift and outcomes after treatment being noted (Mann–Whitney *U*-test, *z* = −0.18, *p* = 0.48).

Kamiryo et al. (21) coregistered stereotactic digital subtraction angiography (DSA) and MEG data to produce superimposed images of AVM angioarchitecture and its relationship to the mapped sensorimotor cortex. This technique, referred to as magnetoencephalographic angiography, was employed in eight patients and demonstrated the vascular relationship of the AVM nidus and feeder vessels to the surrounding functional cortex. With the use of this information, preoperative planning and identification of *en passant* arteries aided in embolization of AVMs, which was performed in five (62.5%) patients. Radiosurgery (*n* = 2) and observation (*n* = 1) were implemented as well.

The use of MEG was successful in detecting cortical changes that ultimately led to modified SRS treatment courses. This is especially important in sparing adjacent tissues that would otherwise be irradiated. However, similar to other mapping modalities, detection of cortical shift by MEG did not confer added clinical benefit with respect to reducing complication rates.

## Discussion

While traditional MR imaging can accurately identify the anatomic relationship of critical brain regions with AVMs, it does not provide information regarding functional connectivity, and it fails to account for the plasticity of brain function that has been shown to occur in the context of underlying brain lesions or injury (22, 23). AVM proximity to eloquent cortex, in addition to nidus size and venous drainage, is a major contributing factor to patient morbidity and postoperative neurologic decline (23). For this reason, accurate mapping and identification of functional reorganization may be important elements in the grading and planning of AVM surgery.

Of the total 12 articles included, functional reorganization was reported to occur in 11 (91.6%) studies. Among these studies, a total of 77 (32.5%) patients experienced cortical reorganization. In two series, SM grading was reported to be directly impacted by the findings of cortical mapping studies (16, 17), with at least half of the studies suggesting preoperative mapping to be useful in identifying eloquent tissue that is otherwise anatomically distorted or unidentifiable on MR imaging. SM grading was downgraded in most cases, as functional eloquence was found to be away from the AVM nidus, favoring surgical resection. These patients did well postoperatively. In addition, functional mapping impacted Gamma Knife dosing and isocenter placement in one study (19), with magnetoencephalographic angiography being useful in preoperative embolization planning in another study (21).

While preoperative planning was modified as a direct result of mapping studies, several studies arrived at the conclusion that cortical reorganization was not statistically different between good and poor functional outcome groups (11–13, 20), despite Lin et al. (12) reporting a trend toward significance. This suggests that translocation of cortical function to different brain regions does not impart a benefit with respect to avoiding neurological deficit postoperatively, and that operation in eloquent regions should be met with continued caution despite evidence of reorganization. This may be due to the fact that not all function has been cortically displaced, with some studies citing continued activation of traditional cortical regions in addition to increased activity in less typical brain areas (12, 14). Cortical reorganization should be viewed and interpreted as a spectrum of change, rather than an all-or-nothing phenomenon.

Three studies evaluated factors impacting cortical reorganization with respect to extent of functional cortical displacement (3, 14, 15). It was reported that greater physical involvement of AVMs within critical cortical regions was more associated with functional reorganization. AVMs located more than one or two gyri away are much less likely to trigger neuroplasticity, with Lee et al. (3) reporting no occurrences of functional reorganization unless there was direct involvement of AVMs with underlying cortical architecture. These findings suggest that cortical mapping may not be indicated for lesions adjacent to, rather than directly involving sensitive brain regions.

It is important to note the heterogeneity of cortical reorganization in AVM. Alkadhi et al. (15) reported three reorganization patterns in their cohort, although there was no clear predictors of which pattern of reorganization would take place for a given lesion. This occasional expansion into nonprimary motor areas further contributes to the unpredictability of neuroplastic changes that take place in AVM, thus favoring the use of fMRI to distinguish among these patterns. This was further demonstrated by Lehéricy et al. (14) where differences in degree of lateralization were noted based on the functional task and corresponding location being evaluated on fMRI. The question remains, however, how to use this information to clinically benefit patients.

There are several limitations to consider when interpreting the findings of these articles. For one, these studies included AVM patients presenting with a spectrum of abnormalities, with some patients presenting asymptomatically or with minor headache, to patients with seizures and marked neurologic deficit, especially in the setting of rupture. As a result, it remains difficult to assess baseline functional organization given that it is unclear if cortical reorganization was present prior to or after the onset of hemorrhage. More consistent with the literature would be that direct hemorrhagic insult to surrounding parenchymal architecture would trigger a neuroplastic response (24–26), although this assumption is complicated by the fact that patients with AVM likely have harbored the lesion since childhood, suggesting that cortical restructuring took place well before hemorrhagic injury. It may be possible that a bleeding AVM further augments preexisting functional reorganization, although this has not been an active area for study. Given that the likelihood of identifying an AVM incidentally remains low, selecting for asymptomatic AVM patients to control for differences in baseline reorganization remains difficult in these studies. However, asymptomatic or incidental cerebral AVMs are being increasingly discovered on cross-sectional imaging studies such as CT and MRI obtained for other reasons. Based on the findings of the ARUBA trial, medical management alone is reasonable for unruptured AVMs, and unnecessary intervention may confer added risk (27). Functional imaging may serve to better determine how to safely evaluate and treat these patients with functional results better than the natural history of the disease.

Another potential limitation lies in the interpretation of fMRI findings given the inherent confounds of this modality. The use of BOLD fMRI sequencing in these studies leaves image acquisition sensitive to hemodynamic perturbations characteristic of AVMs (15). Normally, neurovascular coupling enables active constriction and dilation of microvasculature in the context of changes in neuronal activity (28). In the presence of AVM, regional influences neurovascular coupling impact cerebral blood flow (CBF) and vasoreactivity, having been shown to modify BOLD signaling (9). Additionally, nidus size and volume are significantly associated with total flow (29), suggesting that larger AVMs are more likely to confound fMRI sequencing results. For this reason, AVM angioarchitecture should be assessed and documented carefully when interpreting fMRI findings. The study of Alkadhi et al. (15) was the only study that attempted to account for this hemodynamic susceptibility. Efforts should be made to utilize nonblood-based mapping techniques to avoid these pitfalls, comprising half of our included studies. Other limitations include the small sample sizes of these studies along with the selection bias inherent to retrospective studies. Quantitative analysis was not pursued given the heterogeneity among the patient populations, AVM locations, treatment strategies, and differences in mapping modality and interpretation. Further study is warranted on a larger scale before recommendations can be made regarding the use of fMRI, TMS, or MEG/MSI for preoperatively mapping AVM patients.

## Conclusions

Functional reorganization remains an elusive entity to study, especially in the setting of AVM. Complicated by heterogenous patterns of functional displacement, neuroplasticity in the setting of AVM is dependent on lesion size and location. While factors such as cerebral swelling and mass effect distortion of white matter tracts remain less of a concern, vascular steal phenomenon and abnormal flow dynamics complicate interpretation of fMRI studies not only in the brain region of involvement but also in adjacent tissue structures susceptible to hypoperfusion. For this reason, nonblood-based mapping modalities such as TMS and MEG/MSI are preferred for these lesions. In this systematic review, the authors present the most recent articles presenting case series implementing the use of cortical mapping and its potential benefit in treating patients with AVM. Based on the findings of the included studies, preoperative mapping impacts AVM grading and therefore operative decision making, although how this ultimately affects patient outcomes compared with those without preoperative mapping studies is unclear. In addition, the presence of functional reorganization and its impact on patient outcomes are not apparent, thus warranting further study. Notwithstanding, brain mapping has the potential to be a useful adjuvant in AVM treatment with respect to operative selection and surgical planning.

## Author Contributions

MK: conception. SS, DA, and DB: acquisition of data and drafting the article. All authors critically revised the article and reviewed the submitted version of manuscript. MK approved the final version of the manuscript on behalf of all authors.

## Conflict of Interest

The authors declare that the research was conducted in the absence of any commercial or financial relationships that could be construed as a potential conflict of interest.
